# The complex relationship between depression and dementia

**DOI:** 10.4103/0972-2327.74248

**Published:** 2010-12

**Authors:** Krishna Prasad Muliyala, Mathew Varghese

**Affiliations:** Geriatric Clinic and Services, National Institute of Mental Health and Neurosciences, Bangalore, India

**Keywords:** Alzheimer’s disease, dementia, depression, geriatric, vascular dementia

## Abstract

Dementia and depression are mental health problems that are commonly encountered in neuropsychiatric practice in the elderly. Approximately, half of the patients with late-onset depression have cognitive impairment. The prevalence of depression in dementias has been reported to be between 9 and 68%. Depression has been both proposed to be a risk factor for dementia as well as a prodrome of dementia. This article is a selective literature review of the complex relationship between the two conditions covering definitions, epidemiology, related concepts, treatment, and emerging biomarkers. The methodological issues and the mechanisms underlying the relationship are also highlighted. The relationship between the two disorders is far from conclusive.

## Introduction

Depression, cognitive disturbances, and dementia are some of the commonest mental disorders in the elderly in India. While depression in old age is quite prevalent at about 10–20% of elders depending on the presence of other comorbid physical disorders, the prevalence of dementia is lower than in developed countries at about 3%.[1] The prevalence of milder cognitive disturbances is possibly higher at about 10–15% depending on the definitions of normal senescence to mild cognitive impairment (MCI). The presence of comorbid depression or other physical disorders may cause these prevalence rates to vary depending on whether the cognitive dysfunction is reversible or permanent.[2] In the next 5–10 years, India will have the largest population of elders in the world next only to China. This complex and rather fickle relationship among these three mental disorders would be very challenging for any physician dealing with elders. This paper is a selective literature review using search terms: “depression”, “elderly”, “late-onset depression (LOD)”, and “dementia” in the Pubmed search engine. Important review articles were also used to obtain relevant cross references. The review though not a systematic one attempts to cover most of the issues relevant to these disorders.

### Definitions

A depressive episode or disorder is diagnosed according to ICD-10[3] as a syndrome in the presence of any two of the following: depressed mood, loss of interest and enjoyment, and reduced energy for at least a period of 2 weeks. The episodes are graded as mild, moderate or severe on the basis of number of ancillary and somatic symptoms, and disability. In the literature-related to the topic in discussion, however “depression” has been used in a broader sense. This includes clinically significant depressive symptoms that encompass syndromal depression as defined above in ICD-10 (or the DSM-IV[4]) and also milder depressive symptoms as in subsyndromal depression, nondysphoric depression, and dysthymia. Late life/LOD has been variously defined as a depressive syndrome occurring for the first time after 45–65 years of age.[5]

MCI or minor neurocognitive disorder has been used for an intermediate state between normal cognitive aging and dementia. The precise definition of this potential pre-dementia syndrome continues to evolve. MCI has been further subdivided into amnestic and nonamnestic types: single domain and multidomain.[6] The amnestic type is purported to be a precursor of Alzheimer’s disease (AD) with conversion rates of 10–15% every year.[6]

Dementia is a syndrome due to acquired disease of the brain in which there is a progressive deteriorating disturbance of multiple higher cortical functions such as memory, thinking, orientation, comprehension, calculation, language and judgment in the presence of clear consciousness, sufficient to impair personal activities of daily living[3]. The commonest type of dementia encountered in clinical practice is AD followed by vascular dementia as the next single most frequent cause. Provisional diagnostic criteria for depression in AD have also been proposed by the NIMH.[7]

### Epidemiology

Approximately, half of the patients with LOD have generalized cognitive impairment[8,9] Deficits in executive function and information processing have been described to be typical of LOD. Age, severity of depression, race, education, and vascular risk factors have been noted to make significant contributions to cognitive deficits in LOD.[10]

The cardiovascular health study (CHS) found a cumulative prevalence of 26% for depression among individuals with MCI.[11] In contrast, investigators of the Italian Longitudinal Study on Aging (ILSA) found depressive symptoms in 63% of the patients with MCI.[12] The wide range noted in the two studies might be explained by the use of different instruments to assess depression. The CHS used the Neuro Psychiatric Inventory,[13] whereas the ILSA used the Geriatric Depression Scale (GDS).[14] Both community- and hospital-based studies have shown this wide range of prevalence rates between 9 and 68%. Methodological issues such as use of different definitions of MCI other than sampling may also have contributed to the widely discrepant findings. Across the subtypes of MCI (amnestic vs. nonamnestic; vascular vs. nonvascular), the findings are inconclusive.[15–16] The incidence of depression as observed in the longitudinal studies has been 12 and 30 per 100 person years in hospital and community studies, respectively.[12,18]

The prevalence of depression in dementia has been reported to be 20–60%.[19,20] The prevalence of major depression decreases as severity of dementia increases.[21,22] Vascular and mixed subtypes of dementia have a higher prevalence of depression as compared to AD.[23–25]

### Concepts of pseudo dementia, vascular depression, and apathy

From the clinical standpoint it is necessary to identify the cognitive impairment in depressed patients so also to evaluate for depression in patients with cognitive impairment. In the clinical setting, certain features were believed to be characteristic of depressive pseudo dementia.[26,27] Some of these features described are:

- higher subjective cognitive complaint scores
- complaints of difficulties with concentration and recent memory
- poor effort on examination
- provision of “I don’t know” answers
- inconsistency in sequential performances

This cognitive impairment was considered reversible on treatment with antidepressants or electroconvulsive therapy. These distinctions were, however, not borne out on objective research - patients with dementia being equally likely to give “I don’t know” answers[28] and subjective memory complaints in persons with early stage dementia. In a recent study of 23 centers from low and middle income group countries, subjective memory deficits (SMD) frequency was the lowest in normal elderly subjects (26.2%) and higher in those with depression (50.0%) and dementia (66.7%). Depression and dementia were consistently associated with SMD. In those with dementia, SMD was associated with better cognitive function, whereas the reverse was the case in normal controls[29]

In addition, the persistence of cognitive impairment in a subset of patients after depression was treated prompted commentaries such as these being cases of mistaken identity[30] or “pseudo–pseudo dementia”. It has become increasingly evident that cognitive impairment seen during or after the depressive episode is not “pseudo”, but perhaps a part of depression (state or trait) or a predementia harbinger.[2]

Another concept that is relevant in the context of this complex relationship is that of vascular depression. It was hypothesized that cerebrovascular disease may predispose, precipitate, or perpetuate some geriatric depressive syndromes.[31,32] The interaction and co-morbidity of depression and cerebrovascular disease have been difficult to disentangle. This subset of LOD has greater overall cognitive impairment, apathy, psychomotor retardation, less guilt, greater lack of insight, and greater disability.[33]

Apathy as a neuropsychiatric symptom may be noted in both dementia and depression. Hence, distinguishing isolated apathy from its presentation as a symptom of depression in the context of dementia becomes clinically important. Moreover, nondysphoric depression is commonly observed in patients with dementia.[5] As the severity of dementia increases, the prevalence of apathy increases while that of depression decreases. Apathy may also have a differential impact in the conversion of MCI to dementia with greater conversion in patients having apathy as a symptom.[34] This distinction is difficult to make clinically though it is of therapeutic relevance.

### Rating scales for assessing depression in MCI/dementia and *vice-versa*

The assessment of depression using structured interviews such as structured clinical interview for DSM-IV (SCID) is comprehensive but deemed impractical as it is too lengthy and needs training. They may also not capture features unique to LOD such as apathy and motor impairment. The Geriatric Depression Rating Scale[14] uses a yes or no format and is brief; however, it is only a patient self-report measure of depression and has limitations when used in patients with MCI or dementia. The scale also requires adaptation and modification when used in languages other than English and in rural illiterate population.[35] The Cornell Scale for depression in dementia[36] utilizes clinician’s judgment, patient reports, and informant reports; however, it is insensitive to change. The Neuropsychiatric Inventory[13] assesses 12 behavioral and psychological symptoms on the basis of informant reports and is the most extensively used in the literature though not specific for depression. It can be used by non-psychiatrists and has excellent psychometric properties. Other rating scales used are Hamilton Depression Rating Scale,[37] Beck Depression Inventory,[38] Dementia Mood Assessment Scale,[39] and Centre for Epidemiological Studies for Depression Scale.[40] All the rating scales and structured interview schedules currently available are not without limitations while evaluating for depression in cognitively impaired subjects.

The Mini Mental State Examination (MMSE)[41] is frequently used as a bedside screening tool for cognitive disorders (including dementia), but it suffers from ceiling effects in individuals with MCI. Education and Culturally fair modifications such as the Hindi Mental State Examination (HMSE)[42] also have similar limitations. The Clinical Dementia Rating (CDR) Scale,[43] Mattis Dementia Rating Scale,[44] and the Blessed Orientation–Memory concentration test[45] may be more appropriate in the context of evaluating for cognitive impairment in LOD. In the past years, many instruments which are education and culturally fair like the Cognitive Screening Battery,[46] the 10/66 Dementia Research Group Cognitive test,[47] and the Addenbrooke’s Cognitive Evaluation[48] have been developed in the Indian context for detection of dementia and differentiate from depression. Detailed neuropsychological batteries may be time consuming and are not feasible in routine clinical settings, but may be used for research.

### Biomarkers

Biomarkers in late life mental disorders have involved structural and functional neuroimaging, genomics, CSF studies, neurophysiologic studies, and plasma analysis. We will briefly review select recent literature related to Apo lipoprotein E (*ApoE*) gene and neuroimaging. APOE-4 is a major risk factor for dementia; however, its association with depression has been inconsistent in studies. The Honolulu-Asia Aging Study, a population-based prospective cohort study of Japanese American men examined the independent and combined effects of depression and APOE-4 allele on the risk of dementia and its subtypes. The presence of both depressive symptoms and APOE-4 allele increased several fold the risk for dementia across subtypes.[49] The association of the APOE-4 allele with specific genetic variants of other genes (e.g., CYP2D6, ACE) negatively modulates the therapeutic response to multifactorial treatments affecting cognition, mood, and behavior.[50] Frontal and right parietal white matter hyperintensities were reported as the strongest brain structural correlates of apathy and depression in AD.[51] In patients from a memory clinic, cerebrovascular pathology had no impact on the NPI items in AD.[52] The risk for dementia in patients with depression was observed to be not mediated by smaller hippocampal or amygdalar volumes in the Rotterdam Scan Study.[53] Approximately, one-half of nine remitted patients with LOD demonstrated PiB retention indicative of brain β-amyloid accumulation in cortical areas in a pattern characteristic of early AD in a PET scan study.[54] The gray matter changes in LOD involve smaller volumes of several frontal, temporal areas, cingulum, parahippocampal, inferior parietal area, and putamen. There was correlation of volume loss in these areas with short duration of illness and later age at onset supporting the dementia prodrome model over the toxic stress model.[55]

### Treatment

The treatment of depression occurring in patients with dementia has involved the use of tricyclic agents, SSRIs and MAOIs. The evidence to support the contention that these agents are effective is weak. Randomized controlled trials have evaluated the use of Imipramine, Citalopram, Fluoxetine, Sertraline, and Moclobemide with beneficial results.[56–60] However, a recent multicenter, randomized placebo-controlled trial did not demonstrate efficacy for the treatment of depression with Sertraline in patients with AD. There are on the other hand observations to suggest that antidepressant treatment may reduce cognitive decline in depressed older AD patients.[61] Augmentation of cholinsterase inhibitors with SSRI may improve ADL and global functioning in patients with dementia.[62] There are no RCTs that have evaluated the use of ECT in the treatment of depression in dementia. Nonpharmacological management of depression in the cognitively impaired group involves both patient focused interventions as well as family or caregiver support. A controlled clinical investigation involving the use of two behavioral treatments showed that these methods are effective.[63] Cholinesterase inhibitors ameliorate some types of behavioral symptoms in patients with Alzheimer’s disease. Treatment with donepezil delayed progression to AD among depressed subjects with aMCI.[64]

## Limitations/Caveats While Reviewing Literature

There are certain limitations that one has to bear in mind while reviewing the literature on the complex relationship between depression and dementia.[20] The studies vary in terms of settings—epidemiological/hospital and specialty clinic/general hospital/primary care settings, use of rating scales differs across studies, diagnostic criteria for cognitive impairment are being refined constantly, and the exclusion of subjects with medical comorbidity.[20] There is also a need to operationalize and validate criteria for a depressive syndrome in dementia. In the background of these limitations, inferences drawn from the review of literature may only be inaccurate or erroneous.

## Mechanisms Underlying the Relationship

There is a growing body of evidence to suggest that depression may be a risk factor for the development of dementia.[53,65,66] Past/lifetime history of depression is also known to increase the risk of developing both AD and vascular dementia. This is true even when depression occurred more than 10 years before the onset of dementia.[65] A history of depression nearly doubles the risk of developing dementia—findings of a meta analysis that reviewed both case–control and prospective studies.[67] Interval between diagnoses of depression and AD was positively related to increased risk of developing AD, suggesting that rather than a prodrome, depression may be a risk factor for AD.[68] This is further confirmed by a neuropathological study that demonstrated increased hippocampal plaque and tangle formation in AD patients with lifetime history of depression.[69] Prolonged damage to the hippocampus due to hypercortisolemia linked to depression has been proposed to underlie this finding.[2]

The other way this relationship has been construed is that depression may represent a predementia syndrome or that it serves as a prodrome of cognitive decline.[70] Recent history of depression has been associated with the increased incidence of dementia. Patients with LOD and cognitive impairment go on to develop dementia within a few years after the onset of depression. Depressive syndrome may just be the early manifestation of an underlying neurodegenerative disease. This coexistence can also be because of risk factors that are shared between both disorders such as genetic, vascular, or other environmental determinants.[2,5]

Depression could also be a reaction or a psychological response to the diagnosis of cognitive impairment.[2] Depression can also unmask clinical cognitive impairment. Depression serves to compromise cognitive reserve and allow symptoms of dementia to be manifested earlier than they would have been otherwise.[2] Cognitive impairment in depressed elderly can also be contributed by the use of several tricyclic antidepressants that have anticholinergic effects, though with the use of SSRI’s this should become less likely. All of the above hypotheses are not mutually exclusive and multiple types of interaction may be involved.

## Conclusions

The relationship between depression and dementia is far from clear with the existing body of evidence pointing to a complex interaction. There is a need to sort out several methodological limitations that hinder us from elucidating the relationship. Some of these may include use of uniformed criteria for cognitive impairment, operationalizing, and validating criteria for depression in dementia, using better instruments to measure depression and cognitive impairment when they coexist. This area has enormous public health implications considering our growing elder population, and there is a need to understand the mechanisms involved in the association of these two disorders.
